# Esterification of glycerol from biodiesel production to glycerol carbonate in non-catalytic supercritical dimethyl carbonate

**DOI:** 10.1186/s40064-016-2643-1

**Published:** 2016-06-29

**Authors:** Zul Ilham, Shiro Saka

**Affiliations:** Graduate School of Energy Science, Kyoto University, Yoshida-honmachi, Sakyo-ku, Kyoto, 606-8501 Japan; Institute of Biological Sciences, Faculty of Science, University of Malaya, 50603 Kuala Lumpur, Malaysia

**Keywords:** Glycerol, Biodiesel, Esterification, Glycerol carbonate, Dimethyl carbonate

## Abstract

Conversion of glycerol from biodiesel production to glycerol carbonate was studied by esterification with dimethyl carbonate in a non-catalytic supercritical condition. It was found that in a non-catalytic supercritical condition, glycerol at higher purity gave higher yield of glycerol carbonate at 98 wt% after reaction at 300 °C/20–40 MPa/15 min. The yield of glycerol carbonate was observed to increase with molar ratio, temperature, pressure and time until a certain equilibrium limit. The existence of impurities such as water and remnants of alkaline catalyst in crude glycerol will direct the reaction to produce glycidol. Although impurities might not be desirable, the non-catalytic supercritical dimethyl carbonate could be an alternative method for conversion of glycerol from biodiesel production to value-added glycerol carbonate.

**Electronic supplementary material:**

The online version of this article (doi:10.1186/s40064-016-2643-1) contains supplementary material, which is available to authorized users.

**Graphical abstract:**
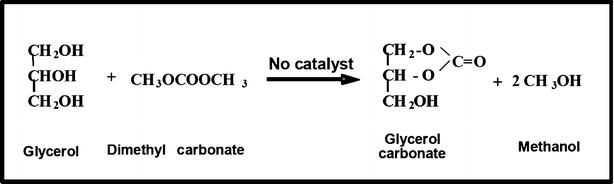
Plausible reaction scheme for conversion of glycerol to glycerol carbonate in non-catalytic supercritical dimethyl carbonate.

## Background

In recent years, growing biodiesel industries have generated a surplus of crude glycerol as the by-product. Approximately 10 wt% of triglycerides could be collected in the form of by-product glycerol from any transesterification of oil with methanol to produce biodiesel (Teng et al. 2014). As the conventional biodiesel plants mostly utilize alkali-catalyzed method, the obtained glycerol is low in purity at 40–70 %, containing remnants of methanol, salt, catalyst residue, soap, fatty acids and glycerides (Ilham and Saka 2012). Without new applications, excess of such crude glycerol could exert impact into refined glycerol market (Yang et al. 2012) and creating a new waste, which could only be used as low-energy fuel for incineration or ruminant feed (Ciriminna et al. 2014). This has prompted the attention of researchers to explore the conversion of the low cost glycerol to value-added products. Several new methods for biodiesel production without producing glycerol in a non-catalytic condition have also been introduced (Ilham and Saka 2010; Saka et al. 2010).

Apart from such works, some researchers also venture into converting glycerol to glycerol carbonate (Algoufi and Hameed 2014; Waghmare et al. 2015; Teng et al. 2016) and other derivatives (Szymanowska-Powalowska 2014; Santacesaria et al. 2010; Ayoub et al. 2012). A heterogeneous alkali-catalyzed esterification of glycerol using waste coal fly ash achieved 96 % glycerol carbonate at 75 °C and 90 min. The catalyst is 4 wt% in loading and could be recycled up to four times (Algoufi and Hameed 2014). Ultrasound-assisted enzyme-catalyst esterification is also feasible but will not work in the existence of impurities such as water and metal ions like sodium, potassium and magnesium in the glycerol (Waghmare et al. 2015). In addition, microwave irradiation has been used for glycerol carbonate production using calcium oxide as catalyst. It was consequently found that 93.4 % of glycerol carbonate yield could be achieved after 5 min reaction time, 1 wt% calcium oxide, 2:1 molar ratio of dimethyl carbonate to glycerol and 65 °C (Teng et al. 2016). However, although microwave irradiation technology might be effective at micro volume, it is known to have limitation at a larger scale.

Glycerol could be a building block to many useful derivatives. Physically, pure glycerol (99 %) is a clear, odorless and hygroscopic liquid under ambient condition. Its boiling point, melting point and flash point are reported to be 290 °C, 18 °C and 177 °C, respectively (Ayoub and Abdullah 2012). The crude glycerol from biodiesel production, on the other hand, is dark brown liquid with faulty smell, being dependent greatly on the methods and the feedstocks used for its conversion (Teng et al. 2014). As purification and distillation of crude glycerol is energy-intensive and economically challenging, one way is to ensure the biodiesel production method that can produce a pure glycerol in the first place such as the supercritical methanol method (Saka and Kusdiana 2001).

This study is, therefore, to report extensively on the esterification of glycerol to glycerol carbonate using non-catalytic supercritical dimethyl carbonate. It should be noted that esterification of glycerol in this study can be, instead, transesterification of dimethyl carbonate by glycerol. This is a matter of which one can be the solvent, dimethyl carbonate or glycerol. Since this work is concerned with the utilization of glycerol, dimethyl carbonate was used as the solvent for its esterification. The route for producing glycerol carbonate was also elucidated to discuss the favorable reaction condition.

## Methods

### Materials

Pure glycerol, dimethyl carbonate, glycerol carbonate, sodium hydroxide (NaOH) and various authentic compounds for standards and chemicals were obtained from Nacalai Tesque Inc., Japan, all of which are of highest purity available. Crude glycerol was also prepared by alkali-catalyzed transesterification (Lubes and Zakaria 2009) and pure glycerol from non-catalytic supercritical methanol (Saka and Kusdiana 2001) for use in this study.

### Characterization of glycerol

Glycerol and methanol contents were analyzed by high performance liquid chromatography (HPLC) LC10-VP System (Column: Ultrahydrogel 120, oven temperature: 40 °C, flow-rate: 1 mL/min, mobile phase: water, detector: RID 10A) (Algoufi and Hameed 2014). Water content in glycerol was determined by Karl-Fischer method in accordance to EN ISO 12937, while standard titration method was used to analyze soap content in glycerol following the American Oil Chemists’ Society (AOCS) Cc 17-95. Salt content in glycerol was determined using precipitation method (Nanda et al. 2014).

### Non-catalytic supercritical dimethyl carbonate esterification of glycerol

**Fig. 1:**
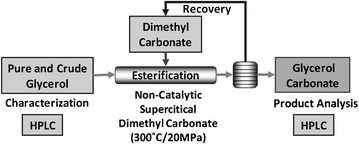
Experimental design depicting non-catalytic supercritical dimethyl carbonate esterification of glycerol

Non-catalytic supercritical dimethyl carbonate esterification of glycerol (pure and crude) was performed in a in a batch-type supercritical biomass conversion system as reported previously (Saka and Kusdiana 2001). Predetermined quantities of glycerol and dimethyl carbonate corresponding to the molar ratio of 1:10 of glycerol to dimethyl carbonate were mixed in a 5 mL Inconel-625 reaction vessel. It was, then, heated to have a reaction at supercritical conditions with dimethyl carbonate (Critical Temperature (Tc): 274.9 °C/Critical Pressure (Pc): 4.63 MPa) by immersing it into a molten tin bath and later quenched into a water bath to stop the reaction. All the experiments and analyses were conducted in compliance with the procedures described in our previous papers (Ilham and Saka 2010, 2012) and the reaction temperature and pressure were monitored by thermocouple and pressure gauge, respectively. Throughout the reaction, pressure was maintained by a custom designed Inconel-625 batch reactor vessel as shown in Additional file 1: Fig. S1. The obtained products were analyzed using HPLC as described beforehand. Experimental design is depicted in Fig. 1. For the calibration curve of the authentic standard products, glycerol and glycerol carbonate were used. All sets of experiments were made to at least triplicate for confirmation of the yields by utilizing 1:10 molar ratio of glycerol to dimethyl carbonate, although not treated statistically. The yield of glycerol carbonate in weight percent as presented in this study refers to the percentage of yields conversion recovered based on theoretical yield. Additional confirmation of glycerol carbonate formation (Additional file 2: Fig. S2) was analyzed using a Bruker AV400 ^1^H NMR spectrometer and referenced to the residual NMR solvent signals. ^1^H NMR (300 MHz, DMSO): *δ* 5.25 (t, ^3^*J*_*HH*_ = 5.6 Hz, 1H), 4.83–4.76 (m, 1H), 4.49 (dd, ^2^*J*_*HH*_ = 8.3, ^3^*J*_*HH*_ = 8.2 Hz, 1H), 4.28 (dd, ^2^*J*_*HH*_ = 8.2, ^3^*J*_*HH*_ = 5.9 Hz, 1H), 3.66 (ddd, ^2^*J*_*HH*_ = 12.6, ^3^*J*_*HH*_ = 5.3, ^3^*J*_*HH*_ = 3.0 Hz, 1H), 3.50 (ddd, ^2^*J*_*HH*_ = 12.7, ^3^*J*_*HH*_ = 5.4, ^3^*J*_*HH*_ = 3.4 Hz, 1H). IR Neat: 1766 cm^−1^ (C = O).

### Conventional esterification

For comparison, the esterification of glycerol by dimethyl carbonate using homogenous NaOH catalyst was carried out in a 250 ml round bottom flask attached to a reflux condenser with a thermometer immersed in a water bath. Predetermined quantities of glycerol and dimethyl carbonate were mixed in molar ratio of 1:5 of glycerol to dimethyl carbonate and heated to 75 °C for 90 min (Ochoa-Gómez et al. 2009). The yields and conversions were determined using similar equations used by Teng et al. (2016).

## Results and discussion

### Properties of pure and crude glycerol

In this study, two grades of glycerol produced by two different biodiesel production methods were used and the properties are shown in Table 1. Properties of commercial glycerol are included just for comparison.

**Table 1:** Properties of glycerol as by-product from different biodiesel production methods

Glycerol from different methods	Purity	Water (wt%)	Salt (wt%)	Soap (ppm)
Alkali-catalyzed glycerol	70	9.82	20.18	35,100
Supercritical methanol glycerol	>98	n.d.	n.d.	n.d.
Commercial glycerol	99	n.d.	n.d.	n.d.

*n.d.* not detected

The properties of crude glycerol produced as by-product from alkali-catalyzed method varied greatly. However, the glycerol produced from non-catalytic supercritical methanol method did not vary, showing similar properties with the commercial glycerol (99 % purity) with no traces of water, salt and soap detectable. This is due to the fact that no catalyst is used in non-catalytic supercritical methanol method (Saka and Kusdiana 2001). On the other hand, crude glycerol from alkali-catalyzed method contains significant amount of water, salt and soap with 70 % purity. Hereinafter in this study, glycerol from alkali-catalyzed method will be referred to as crude glycerol and glycerol from non-catalytic supercritical methanol will be referred to as pure glycerol.

### Glycerol carbonate formation from pure and crude glycerol

**Fig. 2:**
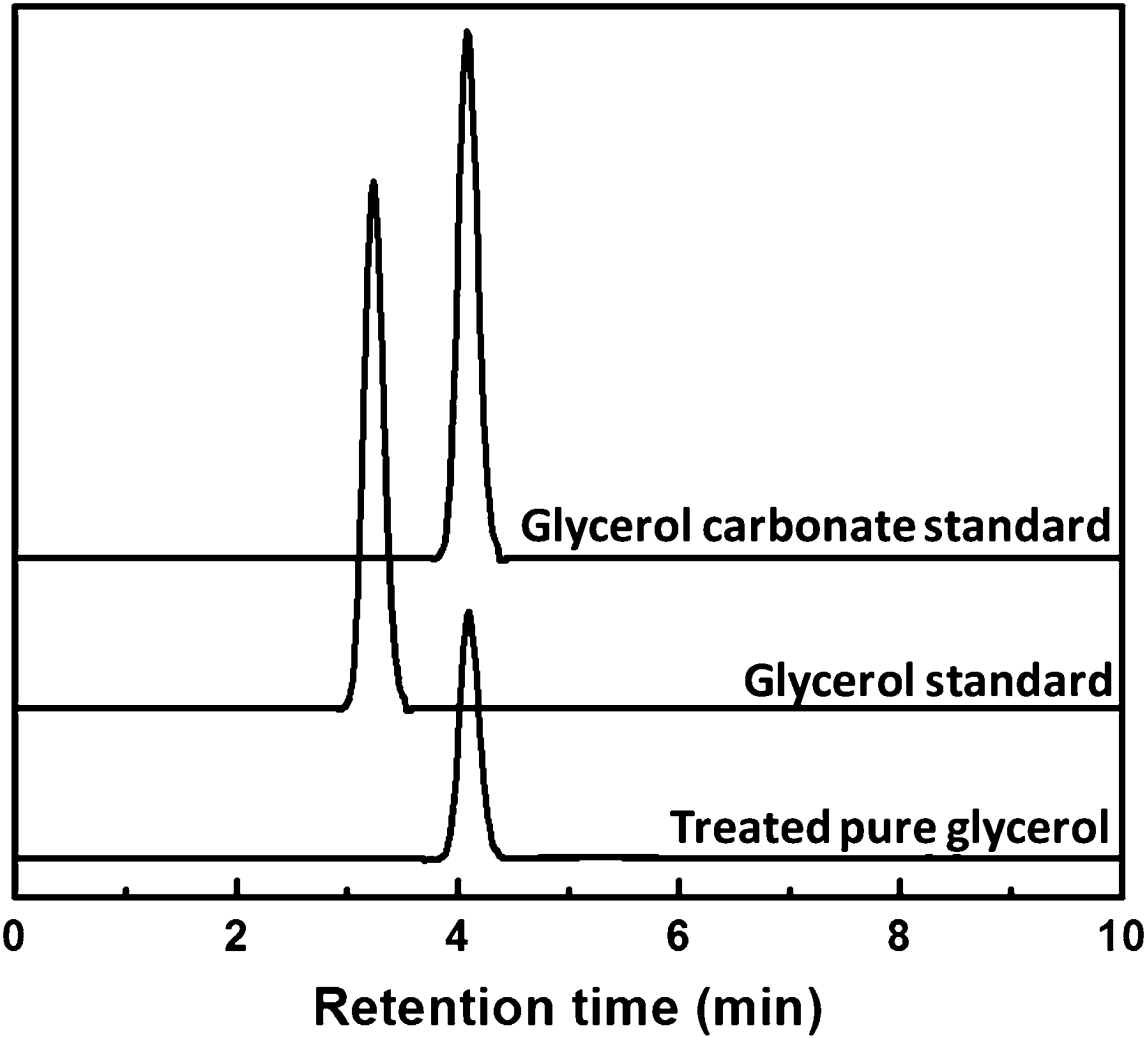
HPLC chromatogram of pure glycerol after treatment in supercritical dimethyl carbonate at 300 °C/20 MPa/15 min without any catalyst applied. Standards of glycerol and glycerol carbonate are shown as authentic compounds

In a trial run, pure glycerol was treated in non-catalytic supercritical dimethyl carbonate at 300 °C/12 MPa for 15 min. When analyzed and compared to several authentic compounds, the resulted compound was found to be glycerol carbonate (4-hydroxymethyl-1,3-dioxolan-2-one), a value-added derivative of glycerol, as depicted at around 4 min retention time in the chromatogram of Fig. 2.

**Fig. 3:**
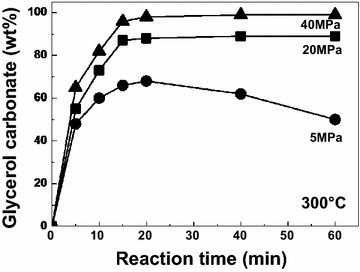
Yield of glycerol carbonate as pure glycerol was treated in non-catalytic supercritical dimethyl carbonate at 300 °C with different reaction pressures

Previously, conversion of glycerol to glycerol carbonate in non-catalytic supercritical dimethyl carbonate was shown to proceed at 300 °C/15 MPa after 15 min with 90 wt% yield (Ilham and Saka 2010). Temperature higher than 300 °C and longer reaction time is not favorable due to the possibility of thermal decomposition (Ilham and Saka 2012). However, Fig. 3 shows the yield of pure glycerol as treated in supercritical dimethyl carbonate at 300 °C with different reaction pressures. It could be seen from the graph that the trend shows higher formation of glycerol carbonate at higher reaction pressure. HPLC plot for partial conversion could be seen in Additional file 3: Fig. S3. Similar trends at lower reaction pressures have been reported (Ilham and Saka 2012). This is in agreement with previous findings in supercritical fluid behavior study where higher reaction pressure always leads to higher yield (Abbott et al. 2005; Warabi et al. 2004).

When the reaction condition was maintained at 300 °C/20–40 MPa, the highest yield of glycerol carbonate based on the theoretical value at 98 wt% could be achieved after 20 min reaction. However, when treated at 300 °C/5 MPa, yield of glycerol carbonate is decreasing particularly after 20 min reaction, indicating possible decomposition of glycerol carbonate under low reaction pressures.

The reaction scheme for the conversion could be expected to proceed in a non-catalytic manner as depicted in the graphical abstract. Glycerol undergoes esterification with dimethyl carbonate, leading to the formation of thermodynamically stable five-member cyclic glycerol carbonate. Methanol, formed from this reaction, was removed by evaporation. The scheme is in agreement with several previous studies describing a similar method in a catalytic manner (Teng et al. 2016; Waghmare et al. 2015; Algoufi and Hameed 2014; Ochoa-Gómez et al. 2009).

### Effect of impurities on glycerol carbonate formation

**Fig. 4:**
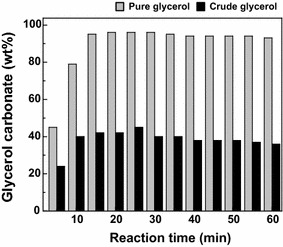
Yield of glycerol carbonate from pure glycerol and crude glycerol when treated in non-catalytic supercritical dimethyl carbonate at 300 °C/20 MPa/15 min

The pure glycerol from supercritical methanol method and crude glycerol from alkali-catalyzed method were both treated in non-catalytic supercritical dimethyl carbonate (300 °C/20 MPa/15 min). As depicted in Fig. 4, pure glycerol showed high conversion to glycerol carbonate, while crude glycerol showed significantly lower yield.

To confirm the effect of impurities in glycerol from alkali-catalyzed method on reducing the yield of glycerol carbonate, a thorough analysis was done on the HPLC chromatogram of products obtained from crude glycerol of alkali-catalyzed method. The chromatogram showed an additional peak. When analyzed and compared with several authentic compounds, this was determined to be glycidol (Ilham and Saka 2012).

**Fig. 5:**
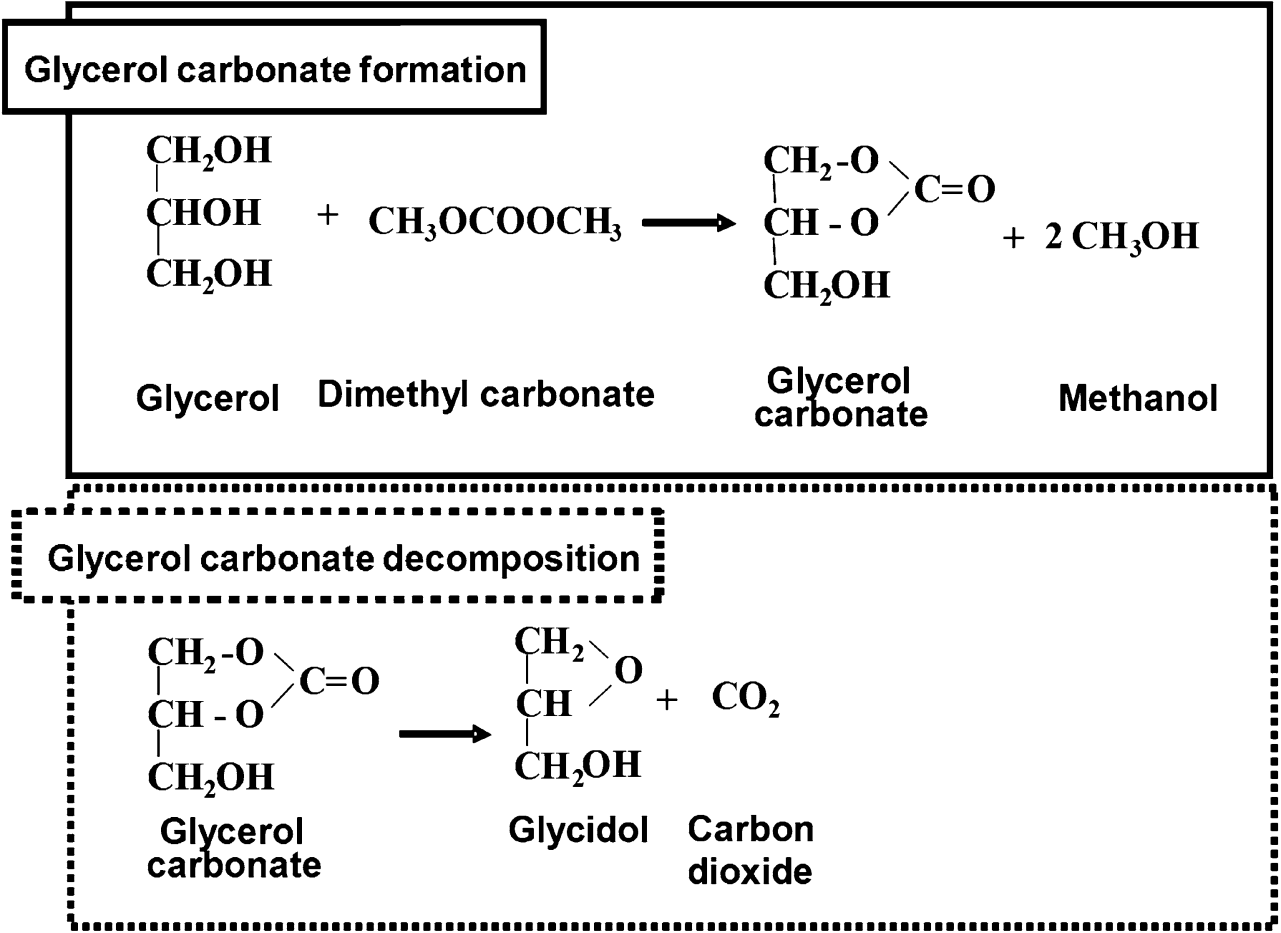
Proposed pathways for glycerol carbonate formation and partial glycerol carbonate decomposition to glycidol and carbon dioxide

Based on these findings, decomposition occurred in the presence of water and salts (impurities in alkali-catalyzed glycerol) to partly decompose glycerol carbonate into glycidol. The proposed scheme for this decomposition pathway is presented in Fig. 5. As discussed beforehand (Fig. 3), low reaction pressures in supercritical condition may also lead to glycidol formation, following a similar pathway as described (Ilham and Saka 2012; Abbott et al. 2005). A research on glycerol carbonates synthesis by coalfly ash catalyst by Algoufi and Hameed (2014) is also in agreement with this finding. Rathore et al. (2014) described the formation of glycerol dicarbonate in their study using dimethyl and diethyl carbonate as reactant but no such intermediate was detected in our study.

### Other methods for glycerol carbonate production

According to Ochoa-Gómez et al. (2009), conventional transesterification by homogeneous NaOH, a range of yield from 35 to 98 wt% could be achieved after 90 min reaction at 75 °C. This might be attractive from an industrial standpoint as a lower reaction time could be used without affecting the yield. However, separation is inevitable and in the case of biodiesel, huge amount of water is needed to wash the dissolved NaOH from the product. In glycerol carbonate production, it is even more complicated as water could not be used. It should be noted that water and glycerol carbonate are miscible. On the other hand, no heterogenous catalyst and acid catalyst could give a high yield of glycerol carbonate (Ochoa-Gómez et al. 2009).

Other possible methods for glycerol carbonate production are ultrasound-assisted lipase-catalyzed method (Waghmare et al. 2015) and microwave-assisted transesterification with calcium oxide (CaO) as catalyst (Teng et al. 2016). Lipase-catalyzed method, similar to other biocatalysis, showed advantages in terms of environmentally-friendly as well as high in yield (>97 wt%), but enzyme is not economical, especially when coupled with high cost ultrasound equipment (Waghmare et al. 2015). Microwave-assisted conversion has been reported to show fast reaction, low operating temperature and effective for crude glycerol but hard to penetrate large volume of sample (Teng et al. 2014). In addition, the use of CaO catalyst is very much dependent on whether or not, it was thermally pre-treated beforehand (Ochoa-Gómez et al. 2009).

Based on the evidence presented beforehand, the non-catalytic supercritical dimethyl carbonate showed a potential as an alternative method for conversion of glycerol to glycerol carbonate. It was found that the supercritical dimethyl carbonate could esterified pure glycerol to glycerol carbonate in high yield without any catalyst applied. When crude glycerol with impurities is used, glycerol carbonate yield decreases in the compensation of glycidol formation. Glycerol carbonate is a stable, colorless liquid currently used industrially as solvent and surfactant (Herseczki et al. 2009), while glycidol is important in the production of epoxy resins and polyurethanes. Its high functionality, together with the versatile and well-investigated reactivity of its hydroxyl functions could supply as a basis for a variety of derivatives (Pagliaro et al. 2007). For comparison, Table 2 shows glycerol carbonate yield and remarks on various glycerol carbonate production technologies.

**Table 2:** Glycerol carbonate yield and remarks on various production technologies

Method	Yield (wt%)	Remark
Alkali-catalyzed	35–98	Separation and purification is needed. Water could not be used as it is miscible with glycerol carbonate and thus, yield decreases
Ultrasound-assisted lipase-catalyzed	>97	Enzyme is costly especially when coupled with ultrasound equipment
Microwave-assisted CaO-catalyzed	93.4	Fast reaction but hard to penetrate large volumes. CaO needs thermal pre-treatment
Non-catalytic supercritical dimethyl carbonate	98	Pure glycerol is needed for high yield but no complicated separation and purification. Use of crude glycerol will lead to glycidol formation

## Conclusions

The results showed that pure glycerol could be converted to value-added glycerol carbonate with a yield of 98 wt% using supercritical dimethyl carbonate (300 °C/20–40 MPa/15 min) without any catalyst applied. On the other hand, when crude glycerol with impurities was used, glycerol carbonate could decompose to glycidol. The high reaction pressure (>10 MPa) must also be maintained in supercritical dimethyl carbonate as low reaction pressure would lead to the decomposition. The formation of value-added chemicals, glycerol carbonate and glycidol from the non-catalytic supercritical dimethyl carbonate method showed the potential of this method to be an alternative way to reduce glycerol glut from biodiesel production.

## Additional files

10.1186/s40064-016-2643-1 Custom designed 5-mL Inconel-625 batch reactor vessel with pressure controller and detector for use into molten tin bath.
10.1186/s40064-016-2643-1 ^1^H NMR spectrum of glycerol carbonate (4-hydroxymethyl-1,3-dioxolan-2-one).
10.1186/s40064-016-2643-1 HPLC plot for partial conversion of pure glycerol in supercritical dimethyl carbonate treatment at 300 °C/20 MPa from 2 min to 20 min reaction time.

## Abbreviations

HPLC: high performance liquid chromatography
AOCS: American Oil Chemists’ Society
DMSO: dimethyl sulfoxide
NMR: nuclear magnetic resonance
