# Supplementary material for: Esterification of glycerol from biodiesel production to glycerol carbonate in non-catalytic supercritical dimethyl carbonate
Source: Springerplus. 2016 Jun 29;5(1):923. doi: 10.1186/s40064-016-2643-1 (PMC4927583; doi:10.1186/s40064-016-2643-1)
Supplement: Supplementary file 1 — 10.1186/s40064-016-2643-1 Custom designed 5-mL Inconel-625 batch reactor vessel with pressure controller and detector for use into molten tin bath. [file 40064_2016_2643_MOESM1_ESM.pdf]

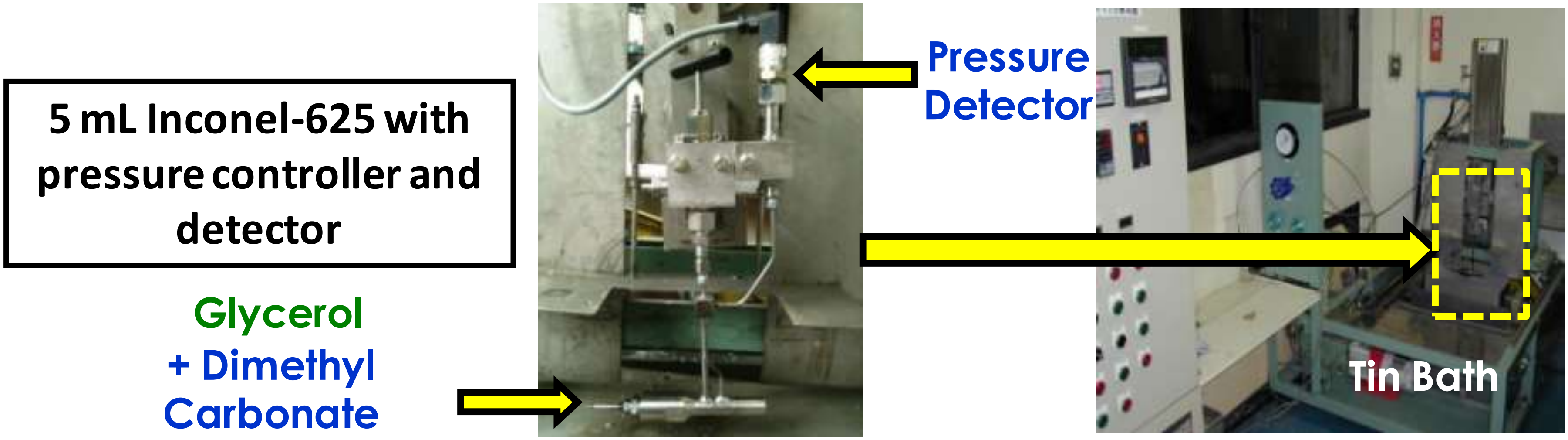

Fig. S1. Custom designed 5-mL Inconel-625 batch reactor vessel with pressure controller and detector for use into molten tin bath
