# Supplementary figures and images for: Esterification of glycerol from biodiesel production to glycerol carbonate in non-catalytic supercritical dimethyl carbonate
Source: Springerplus. 2016 Jun 29;5(1):923. doi: 10.1186/s40064-016-2643-1 (PMC4927583; doi:10.1186/s40064-016-2643-1)

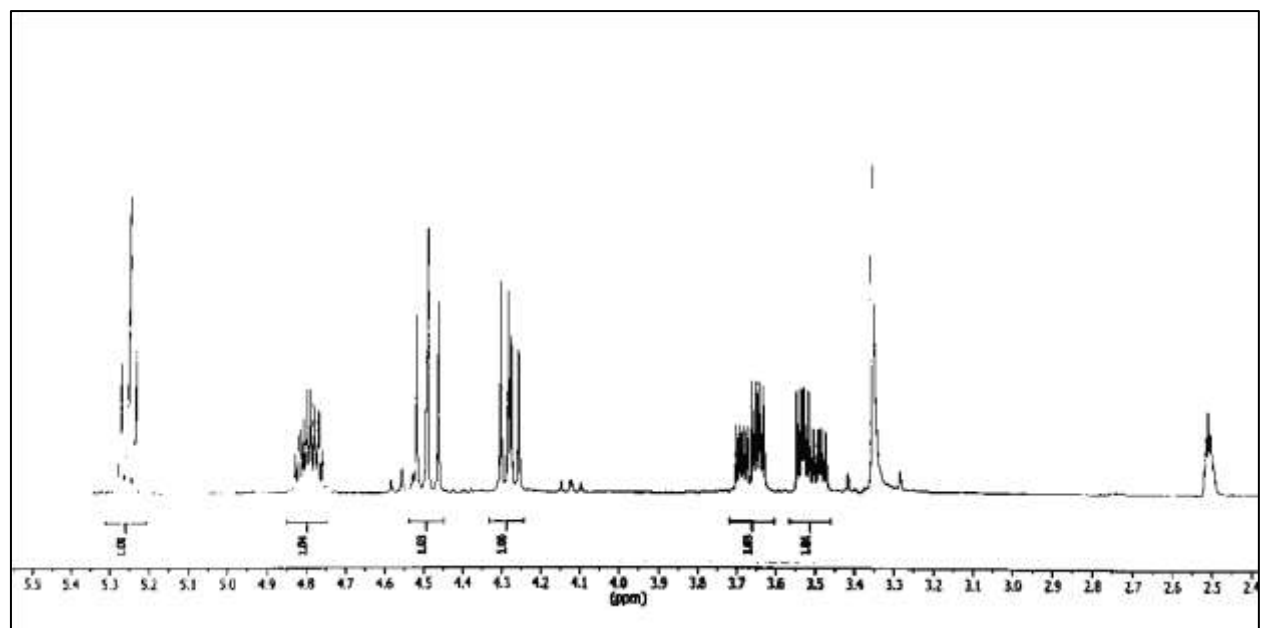

**Fig. S2.**  $^1\text{H}$  NMR spectrum of glycerol carbonate (4-hydroxymethyl-1,3-dioxolan-2-one)

Supplement: Supplementary file 2 — 10.1186/s40064-016-2643-1 1H NMR spectrum of glycerol carbonate (4-hydroxymethyl-1,3-dioxolan-2-one). [file 40064_2016_2643_MOESM2_ESM.pdf]
