# Supplementary material for: Esterification of glycerol from biodiesel production to glycerol carbonate in non-catalytic supercritical dimethyl carbonate
Source: Springerplus. 2016 Jun 29;5(1):923. doi: 10.1186/s40064-016-2643-1 (PMC4927583; doi:10.1186/s40064-016-2643-1)
Supplement: Supplementary file 3 — 10.1186/s40064-016-2643-1 HPLC plot for partial conversion of pure glycerol in supercritical dimethyl carbonate treatment at 300 °C/20 MPa from 2 min to 20 min reaction time. [file 40064_2016_2643_MOESM3_ESM.pdf]

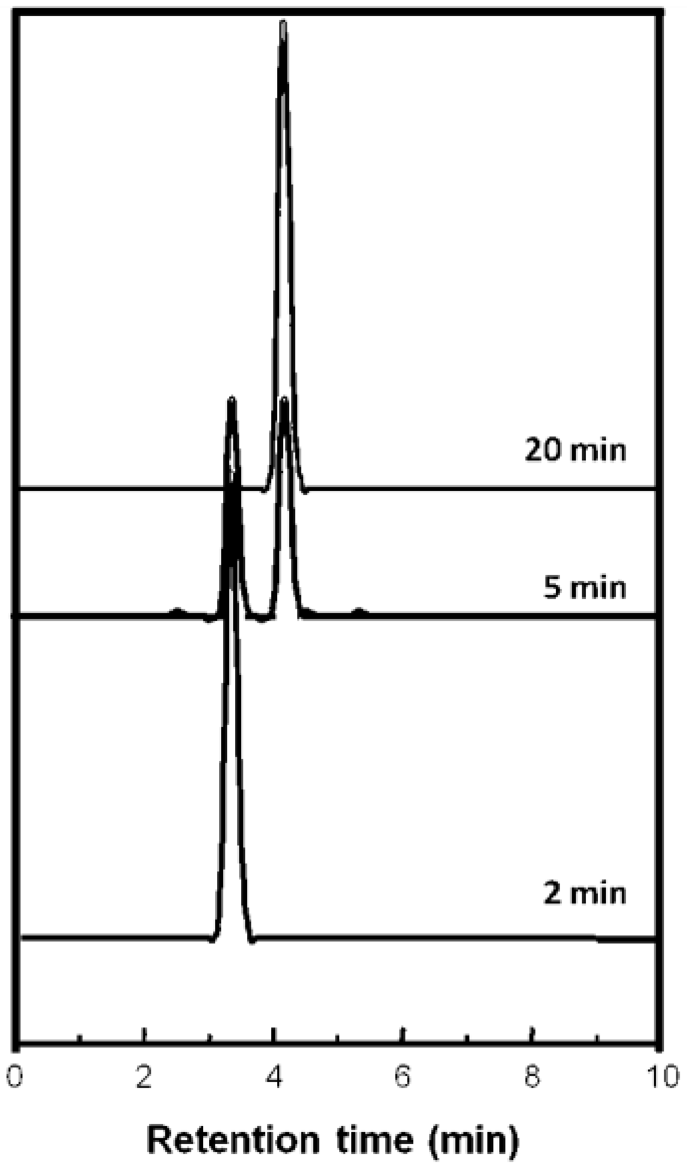

Fig. S3. HPLC plot for partial conversion of pure glycerol in supercritical dimethyl carbonate treatment at 300°C/20MPa from 2 min to 20 min reaction time.
